# Detection and Continuous Tracking of Breeding Pigs with Ear Tag Loss: A Dual-View Synergistic Method

**DOI:** 10.3390/ani15192787

**Published:** 2025-09-24

**Authors:** Weijun Duan, Fang Wang, Honghui Li, Na Liu, Xueliang Fu

**Affiliations:** 1 College of Computer and Information Engineering, Inner Mongolia Agricultural University, Hohhot 010018, China; 2 National Center of Technology Innovation for Dairy-Breeding and Production Research Subcenter, Hohhot 010018, China; 3 Key Laboratory of Smart Animal Husbandry at Universities of Inner Mongolia Autonomous Region, Hohhot 010018, China; 4 College of Animal Science, Inner Mongolia Agricultural University, Hohhot 010018, China

**Keywords:** ear tag loss detection, channel pruning, dual-view synergy, individual tracking, motion attention

## Abstract

The lossof ear tags in breeding pigs can lead to the loss or confusion of individual identity information. Timely and accurate detection, along with continuous tracking of breeding pigs that have lost their ear tags, is crucial for improving the precision of farm management. However, considering the real-time requirements for the detection of ear tag-lost breeding pigs, coupled with tracking challenges such as similar appearances, clustered occlusion, and rapid movements of breeding pigs, this paper proposed a dual-view synergistic method for detecting ear tag-lost breeding pigs and tracking individuals. First, a lightweight ear tag loss detector was developed by combining the Cascade-TagLossDetector with a channel pruning algorithm. Second, a synergistic architecture was designed that integrates a localized top-down view with a panoramic oblique view, where the detection results of ear tag-lost breeding pigs from the localized top-down view were mapped to the panoramic oblique view for precise localization. Finally, an enhanced tracker incorporating Motion Attention was proposed to continuously track the localized ear tag-lost breeding pigs. Experimental results indicated that, during the ear tag loss detection stage for breeding pigs, the pruned detector achieved a mean average precision of 94.03% for bounding box detection and 90.16% for instance segmentation, with a parameter count of 28.04 million and a detection speed of 37.71 fps. Compared to the unpruned model, the parameter count was reduced by 20.93 million, and the detection speed increased by 12.38 fps while maintaining detection accuracy. In the tracking stage, the success rate, normalized precision, and precision of the proposed tracker reached 86.91%, 92.68%, and 89.74%, respectively, representing improvements of 4.39, 3.22, and 4.77 percentage points, respectively, compared to the baseline model. These results validated the advantages of the proposed method in terms of detection timeliness, tracking continuity, and feasibility of deployment on edge devices, providing significant reference value for managing livestock identity in breeding farms.

## Simple Summary

Ear tags are widely used as the primary method for individual identification in breeding pigs. However, the loss of ear tags can disrupt production records, compromise health management, and create confusion in pedigree information, ultimately undermining genetic selection and precise management efforts. Simply detecting lost ear tags is not enough, as breeding pigs in large pens often look very similar, move frequently, and have ears that are frequently obscured from the staff’s sight, making it challenging for farm staff to promptly and accurately identify the affected animals. To address these challenges, we present an integrated detection–mapping–tracking framework that combines a localized top-down perspective with a panoramic oblique view. Our experimental results demonstrate that, while remaining lightweight, the proposed framework can automatically detect and continuously track breeding pigs that have lost their ear tags, even in complex farm environments. This enables staff to quickly identify loss events, accurately locate individual animals, and efficiently reapply new ear tags. As a non-invasive solution, our approach offers strong technical support for the automated monitoring and management of ear tag status in precision livestock farming and holds promise for application to cattle, sheep, and other livestock species.

## 1. Introduction

In large-scale intelligent breeding farms, ear tags serve as the primary means for identifying individual pigs, with their corresponding numbers linked to critical information such as production records, health and vaccination histories, and genetic breeding data [1]. However, factors such as ear tag material degradation, mutual biting between pigs, and friction with facilities increase the risk of ear tag loss. The loss of ear tags can lead to significant consequences for production management and genetic breeding efforts due to the resultant loss or confusion of breeding pigs’ identity information [2]. Therefore, timely and accurate detection of pigs that have lost their ear tags, along with continuous tracking, enables farm staff to quickly identify ear tag loss incidents, accurately locate the affected breeding pigs, and promptly reattach new tags. This process is vital for enhancing individual identity management in smart farming and improving the overall standards of precision livestock management.

Previous studies have reported that the ear tag loss rate in pigs throughout their life cycle ranges from 0.2% to 19.4%, with an average of 2.8%. Notably, no significant differences have been observed between different types of ear tags [3]. In this study, field surveys conducted at pig breeding farms revealed that manual observation remains the primary method for detecting ear tag loss in breeding pigs. However, challenges such as limited observation angles, high visual similarity, individual occlusion, and rapid movement make it particularly difficult to detect and track pigs that have lost their ear tags, resulting in high labor intensity, substantial staffing demands, and excessive time consumption. Furthermore, some farms have introduced monitoring methods based on RFID ear tags and channel devices [4,5]. However, these methods require pigs to pass through designated channels, which not only fails to meet real-time detection requirements but also increases the workload associated with additional management tasks.

In recent years, the rapid advancement of computer vision technology has enabled significant progress in pig individual identification [6,7,8,9], posture classification [10,11], behavior recognition [12,13,14,15], and target tracking [16,17,18]. These advancements provide a strong foundation for the automated detection and tracking of pigs that have lost their ear tags.

In the field of ear tag loss detection, Wang et al. (2023) conducted the first study targeting breeding pigs [2]. They proposed an enhanced Cascade Mask R-CNN model that detects pigs and ear tags separately from a top-down view and determines ear tag loss by calculating the intersection area of their masks, achieving a detection accuracy of 92.86%, albeit with a 27.9% increase in model size. Building on this, Wang et al. (2024) developed the Cascade-TagLossDetector, which integrated a lightweight convolutional unit (IRDSC) and was evaluated on a self-constructed instance segmentation dataset containing pigs both with and without ear tags [19]. This method reached 90.02% detection accuracy while reducing the model size by 14.13%. More recently, Duan et al. (2025) proposed Adapt-Cascade, a motion blur-aware multi-scale framework for ear tag loss detection in breeding pigs, achieving 93.46% accuracy at 19.20 frames per second [20]. Although these methods demonstrate promising detection accuracy, their inference speed remains insufficient for real-time applications in production environments. Consequently, reducing model size while maintaining accuracy continues to be a critical challenge.

In the domain of pig tracking, Wutke et al. (2021) proposed a method based on top-down keypoint detection and Kalman filtering, achieving continuous tracking and contact detection for approximately 10 pigs [21]. Tu et al. (2024) introduced the RPTrack framework for complex pigsty environments, which improved multi-target tracking accuracy under varying lighting conditions with up to 12 pigs [22]. Mora et al. (2024) combined BoT-SORT tracking with RFID ear tag information to enable continuous tracking in top-down videos of 12 pigs, effectively mitigating identity drift caused by visual similarity [23]. However, these approaches generally rely on top-down views, and experimental settings are typically confined to small pigpens with a limited number of animals. In actual production, the top-down view cannot fully cover large-scale pigsties, making continuous facility-wide tracking unfeasible. Moreover, although some studies have addressed appearance similarity and lighting variations, maintaining consistent identity and achieving robust tracking remain major challenges in large-scale environments characterized by rapid movement and frequent occlusions.

This paper presents, for the first time, a dual-view synergistic method for detecting and continuously tracking breeding pigs that have lost their ear tags in production environments. Specifically, pigs frequently visit the feeding area of the pigsty, where ear tags are almost fully visible from a top-down view. Thus, the top-down view is employed for ear tag loss detection. However, since this localized perspective cannot cover the entire pigpen, a panoramic oblique view is additionally introduced to enable continuous tracking of pigs that may lose their ear tags. The proposed method supports both detection and localization of pigs with ear tag loss, thereby providing a practical and efficient technical solution for the timely reapplication of ear tags. The main contributions of this study are as follows:

(1) We leverage the high-precision detection capability of the Cascade-TagLossDetector in production environments, where pigs are classified into tagged and untagged categories. By integrating a channel pruning algorithm, we propose a lightweight detector, Cascade-TLDP, that achieves a balance between detection accuracy and inference speed.
(2) We develop a synergistic mapping method that combines localized top-down and panoramic oblique views. Using the detection bounding boxes of pigs with ear tag loss from the top-down view as input, this method performs position mapping in the panoramic oblique view to effectively localize untagged pigs.
(3) We introduce the STARK-MOT tracker with Motion Attention, which performs motion weighting in both spatial and temporal domains. This mechanism guides the encoder to prioritize motion features during multi-frame feature encoding, thereby improving modeling and tracking performance in complex scenes and enabling continuous tracking across the entire visual field.
(4) We provide robust technical support for building a real-time, efficient, and automated system that preserves individual identity and facilitates dynamic management in pig farms, thereby significantly enhancing intelligent farm management.

## 2. Materials and Methods

### 2.1. Dataset

#### 2.1.1. Data Acquisition

**Figure 1:**
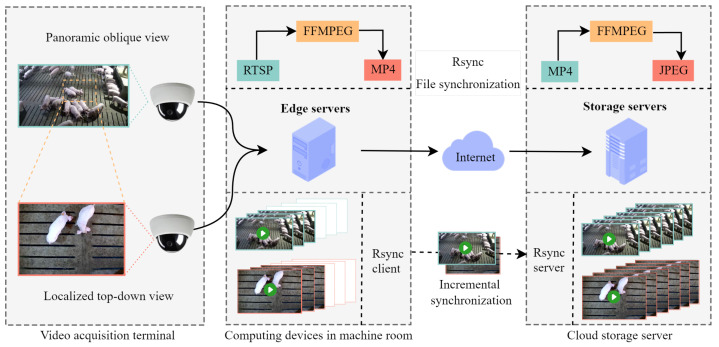
The architecture of the data acquisition system. RTSP is a streaming protocol that can obtain audio–video streams directly from cameras; solid arrows indicate data transmission direction; dotted arrows indicate incremental data synchronization.

This study was conducted at a large-scale breeding pig farm located near Hohhot City in the Inner Mongolia Autonomous Region of China. The core facilities of the farm include eight replacement gilt houses (64 pens in total), four gestation houses (with more than 4600 gestation stalls), and 17 farrowing houses (952 farrowing crates), collectively housing 5500 breeding pigs. Replacement gilts are housed in spacious pens, each containing about 35 pigs. Due to their frequent interactions and high activity levels, ear tag loss was particularly prevalent among these animals. For data collection, a cloud–edge dual-view system was installed in three replacement gilt pens. The system consisted of video acquisition terminal, edge computing devices in the machine room, and a cloud storage server. The overall data acquisition workflow is illustrated in Figure 1.

(1) Video acquisition terminal: Each pilot gilt pen measures 5.3 × 6.0 m and houses 35 breeding pigs, including 5 without ear tags and 30 with ear tags. Two DS-2PT7D20IW-DE dome cameras (Hikvision, Hangzhou, China) were installed in each pen. The cameras provide a resolution of 1920 × 1080 pixels at 25 frames per second. One camera was ceiling-mounted 3.4 m above the feeding area to capture high-definition top-down data for ear tag loss detection, effectively highlighting the ear region. The second camera was suspended at the midpoint above one side of the pen to capture panoramic data for continuous tracking. Both cameras automatically switched between day and night modes by detecting illumination intensity, enabling the collection of color and grayscale video data under varying activity states during both daytime and nighttime.

To ensure experimental rigor and reproducibility, all selected pigs were 2–3-month-old female Landrace gilts in good physical condition, without apparent health abnormalities or clinical diseases. All pigs were reared under identical housing conditions and stocking densities, with consistent feeding, watering, and environmental parameters. Management practices were standardized, and no special treatments were applied. Data were collected from October to December 2022. Raw video data under varying light conditions and activity states were obtained through 24-h continuous dual-view monitoring of pigs within the gilt pens.

(2) Edge computing devices in the machine room: The edge devices in the machine room were responsible for local data storage, compression, and forwarding. Using FFMPEG (v5.1) for real-time transcoding, RTSP streams were converted into MP4 files, with a new file generated every ten minutes. The incremental data were then synchronized to the cloud storage server via RSYNC (v3.2.4).

(3) Cloud storage server: The cloud server received and stored incremental video data transmitted from the edge devices through RSYNC, from which the data could be retrieved for experimental use.

#### 2.1.2. Data Preprocessing

**Figure 2:**
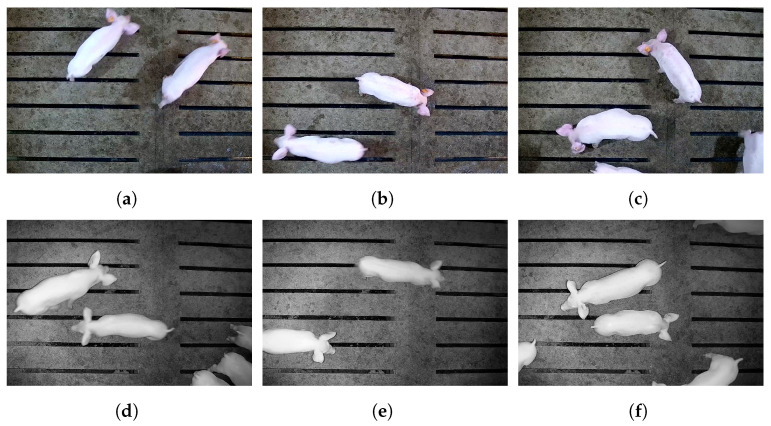
Examples of ear tag loss detection images under different activity states and lighting conditions. (**a**) Daytime active; (**b**) daytime mixed state; (**c**) daytime stationary; (**d**) nighttime active; (**e**) nighttime mixed state; (**f**) nighttime stationary. In a single image, active pigs exhibit noticeable motion blur or ghosting; stationary pigs show no motion blur; mixed-state images contain both active and stationary pigs.

This study adopted a timestamp alignment method to align dual-view data. A total of 782 paired video files containing pigs with ear tag loss were extracted simultaneously from top-down and panoramic views. Each video lasted 1–3 min and captured various activity states of pigs during both daytime and nighttime. Frames were sampled at 1 frame per second. To reduce overfitting caused by redundant images, the Structural Similarity Index (SSIM) algorithm [24] was used for image filtering. The SSIM threshold was set to 0.78, based on multiple experiments with this dataset and supported by relevant studies [2,19]. This threshold effectively removed redundant images while preserving data diversity. Ultimately, 6752 images were retained, including 1403 daytime active, 2112 daytime mixed-state, 1893 daytime stationary, 313 nighttime active, 547 nighttime mixed-state, and 484 nighttime stationary images. Representative examples are shown in Figure 2.

#### 2.1.3. Dataset Construction

(1) The detection dataset was annotated at the instance level using EISeg (v1.1) and divided into training and test sets with an 8:2 ratio. The dataset contained images of breeding pigs under different lighting and motion conditions, ensuring that the distribution of images across scenes accurately reflected real-world scenarios. This approach provided sufficient sample size and a reasonable data split. The training set included 5404 images, comprising 5185 pig instances with visible ear tags and 7261 with non-visible ear tags, while the test set contained 1348 images, including 1396 pig instances with visible ear tags and 1848 with non-visible ear tags. Here, “non-visible ear tags” includes cases where ear tags are outside the camera frame, and not just ear tag absence.

(2) The individual tracking dataset was annotated using DarkLabel (v2.4) in the MOT-17 format. Each annotation recorded the target ID, the coordinates of the upper-left corner of the bounding box, and its width and height. The annotations were then exported as tracking trajectory data and converted into the LaSOT format for single-target tracking. In total, 2968 complete pig tracking trajectories were obtained and split into training and test sets with an 8:2 ratio. The data distribution is summarized in Table 1.

### 2.2. Methods

**Figure 3:**
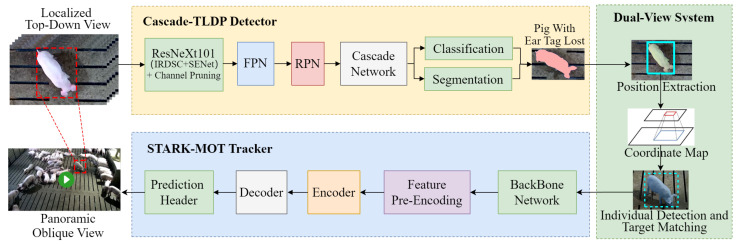
The architecture of the dual-view synergistic system for detecting and tracking pigs with ear tag loss. Top-down view images from the feeding area are first processed by Cascade-TLDP to detect pigs with ear tag loss. The detected bounding boxes are then integrated into the dual-view synergistic module for position mapping in the panoramic oblique view, where continuous tracking is performed using STARK-MOT.

In this study, we propose a dual-view synergistic lightweight method for detecting and tracking breeding pigs with ear tag loss. The framework consists of three components: a detector for pigs with ear tag loss, a dual-view synergistic system, and a tracker for pigs with ear tag loss, as illustrated in Figure 3. First, a lightweight detector named Cascade-TLDP was developed by integrating the Cascade-TagLossDetector with a channel pruning algorithm. This detector classifies pigs in localized top-down view images of the feeding area as either with visible ear tags or with non-visible ear tags, depending on tag visibility and whether the pig is fully within the frame. During training, Cascade-TLDP ranked channel importance using channel information entropy, selected candidate channels for pruning, and removed redundant channels at a pruning rate of 50%. The pruned lightweight backbone replaced the original network, enabling efficient and accurate detection of pigs with ear tag loss. Second, a dual-view synergistic system was constructed, in which the detection bounding boxes from Cascade-TLDP served as inputs. Through camera calibration, coordinate transformation, and target matching between the localized top-down view and the panoramic oblique view, the positions of pigs with ear tag loss were determined within the panoramic oblique view. Finally, an enhanced STARK-MOT tracker with Motion Attention was designed. Built upon the STARK framework [25], it models global spatiotemporal dependencies in video sequences to capture state changes of pigs with ear tag loss. The Motion Attention mechanism reinforces the model’s focus on motion regions, thereby enabling continuous tracking of localized pigs with ear tag loss in the panoramic oblique view.

#### 2.2.1. Cascade-TLDP Detector

**Figure 4:**
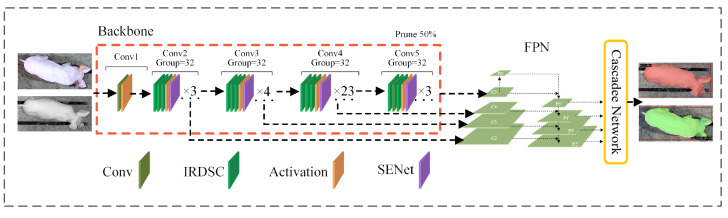
The structure of the Cascade-TLDP detector. It consists of a backbone network (ResNeXt101 with IRDSC modules and SENet channel attention), a feature pyramid network (FPN), and a cascade detection network. “Prune 50%” indicates structured pruning of 50% of the channels based on BatchNorm scaling factors.

Although the Cascade-TagLossDetector developed by our team previously achieved high detection accuracy for pigs with ear tag loss, its network structure contains redundant feature channels, resulting in an excessive number of parameters. This not only increases computational overhead during training and inference but also limits deployment and operational efficiency in resource-constrained environments, such as edge devices. To meet the real-time requirements for detecting and tracking pigs with ear tag loss in production scenarios, this paper proposes Cascade-TLDP, a detector for pigs with ear tag loss that integrates channel pruning methods into the Cascade-TagLossDetector, as illustrated in Figure 4. The proposed detector consists of a backbone network, a region proposal network, and a cascade detection network.

(1) Backbone Network: ResNeXt101 was used as the backbone for feature extraction. A feature extraction module, IRDSC, was constructed by integrating depthwise separable convolutions with inverted residual structures, replacing the standard convolutions in ResNeXt101. This design enhanced feature extraction capability while reducing computational complexity. In each grouped convolution layer of ResNeXt101, the SENet channel attention mechanism was incorporated to capture correlations between channels. This mechanism improved the representation of salient features and enabled adaptive adjustment based on channel importance. As a result, the model achieved a bounding box mAP of 94.15% and a Mask mAP of 90.32%, with a detection speed of 25.33 FPS, which was still insufficient for real-time detection.

To mitigate redundancy in certain channels that contributed little information to the final output, a structured pruning method was introduced. By selectively pruning channels according to their importance, this method reduced the parameter count and computational complexity while maintaining accuracy, thereby improving detection speed.

Channel importance was measured using the BatchNorm scaling factor γ. During backbone training, an $L_{1}$ regularization term was added to encourage γ values to converge towards a sparse distribution, as shown in Equation (1). This design effectively compressed the weights of unimportant channels towards zero, while retaining larger values for informative channels, thus improving discrimination in channel selection.

(1) $$L_{\mathrm{total}}=L_{\mathrm{cascade}}+\lambda \sum_{l=1}^{L}\;\gamma^{\left(l\right)}\;$$

where $L_{\mathrm{cascade}}$ denotes the training loss of the Cascade-TagLossDetector; λ is the balance coefficient for sparse regularization; and $\gamma^{\left(l\right)}$ represents the scaling factor of the BatchNorm layer at the *l*-th layer.

Next, the channels of each layer were sorted in ascending order of their γ values. Channels with lower importance were pruned under different pruning rates, simultaneously removing the associated convolutional kernels and BatchNorm parameters. After pruning, the number of input and output channels in the convolutional layers was updated, and the BatchNorm configurations were reconstructed. Following Yu et al. (2022) [26], the impact of different sparsity rates on model performance was analyzed. Under a pruning rate of 50%, an optimal balance between model size and detection accuracy was achieved.

(2) Region Proposal Network: The region proposal network (RPN) generated anchor boxes for candidate target regions using the four 256-channel multi-scale feature layers of the feature pyramid network. By mapping these anchor boxes back to the original image space, candidate regions for subsequent detection and segmentation were obtained.

(3) Cascade Detection Network: The cascade detection network employed cascade detection and regression branches to identify target objects and refine their coordinates. This process optimized classification and regression loss calculations by leveraging the advantages of Focal Loss for minority categories. As a result, the model placed greater emphasis on minority samples and hard-to-detect cases. This approach effectively improved detection accuracy, particularly under nighttime conditions and in scenarios characterized by imbalanced positive and negative sample distributions in the training dataset.

In this study, the dataset for detecting pigs with ear tag loss was classified based on ear tag visibility, distinguishing between pigs with visible ear tags and those with non-visible ear tags. Non-visible ear tags arose in two scenarios: (i) pigs with ear tag loss, and (ii) pigs whose ear tags were outside the image field of view. The latter case could not be conclusively identified as ear tag loss. Therefore, this study defined the identification criteria for pigs with ear tag loss as follows: if the ear tag was not visible and the predicted mask was at least one pixel away from the image boundary, the pig was classified as having ear tag loss. In other words, if a breeding pig was fully contained within the field of view and detected as having a non-visible ear tag, it was labeled as a pig with ear tag loss, and the coordinates of its detection box were recorded.

#### 2.2.2. Dual-View Synergistic Method

In this study, we achieve the position mapping of two cameras from both a localized top-down view and a panoramic oblique view through the processes of camera calibration [27], coordinate transformation, and target matching.

(1) Camera Calibration: This study utilizes the slatted floor as the reference plane (Z=0) for the world coordinate system. By measuring the three-dimensional positions (X,Y,Z) and rotation angles of both the localized top-down view and global oblique view cameras relative to this coordinate system, we can determine the initial values of the external parameters. Subsequently, several representative feature points are selected on the slatted floor. The three-dimensional world coordinates are measured manually, and their corresponding two-dimensional pixel coordinates are annotated in the synchronously captured localized top-down and global oblique view images to complete the camera calibration.

During calibration, the extrinsic parameters, R and T, are initially established using physical measurement results. Subsequently, known 3D points are projected onto the image plane to compute the reprojection error between predicted pixel locations and their manually annotated counterparts. By minimizing the reprojection error for all feature points across both viewpoints, the intrinsic matrix *K* and the extrinsic parameters R,T are jointly optimized to yield more accurate camera parameters. This projection relationship is articulated in Equation (2), while the optimization objective function is presented in Equation (3).

(2) $$s\cdot \left(\begin{array}{c} u\\ v\\ 1 \end{array}\right)=K\cdot \left[R|T\right]\cdot \left(\begin{array}{c} X\\ Y\\ Z\\ 1 \end{array}\right)$$

where, (u,v) denotes pixel coordinates, (X,Y,Z) represents the world coordinates of the feature point on the slatted floor (Z=0), *K* is the internal parameter matrix, [R|T] is the external parameter matrix.

(3) $$E=\sum_{i=1}^{N}\sum_{j=1}^{2}{∥\left(\begin{array}{c} u_{ji}\\ v_{ji} \end{array}\right)-\pi \left(K_{j},R_{j},T_{j},X_{i}^{w}\right)∥}^{2}$$

In this context, $\left(u_{ji},v_{ji}\right)$ denotes the pixel coordinates of the *i*th point in the *j*th camera, $X_{i}^{w}$ represents the known 3D world coordinates. The projection operator is denoted as π(·), $K_{j},R_{j},T_{j}$ refer to the internal and external parameters that require optimization. These parameters are determined using the Levenberg–Marquardt nonlinear least-squares method, which ultimately facilitates an accurate spatial mapping between the world coordinate system and the image plane.

(2) Coordinate transformation: The target frames of breeding pigs with ear tag loss, as detected by Cascade-TLDP, were transformed into the panoramic oblique view coordinate system using rotation (R) and translation (T), as shown in Equation (4).

(4) $$P_{2}=R\cdot P_{1}+T$$

In the context, $P_{1}$ refers to the coordinates of the target bounding box detected from a top-down perspective, and $P_{2}$ denotes the corresponding point coordinates in the panoramic view following coordinate transformation.

(3) Individual pig detection: In the panoramic oblique view, Cascade-TLDP was used to detect individual pigs, generating bounding boxes for each breeding pig.

(4) Target matching: The normalized Euclidean distance metric was employed to compute the distance between $P_{2}$ and the center points of all detected bounding boxes in the frame. Candidate boxes were then selected according to the nearest-neighbor principle. To improve robustness, a normalized distance threshold was applied to filter detection results. The system only outputs the identification result for pigs with ear tag loss if the minimum distance is smaller than the threshold; otherwise, no valid match is considered. Ultimately, the identified pig with ear tag loss is marked in the panoramic oblique view.

#### 2.2.3. STARK-MOT Tracker

In this study, when Cascade-TLDP detected pigs with ear tag loss, the system immediately activated the tracker and triggered an alert to notify farm staff for timely re-tagging. Previous studies have reported that the average rate of ear tag loss in pigs over their life cycle is 2.8% [3]. By enabling timely re-tagging, the likelihood of multiple pigs with ear tag loss occurring simultaneously within a pigsty was significantly reduced. Multi-target tracking schemes typically rely on real-time multi-target detection models, which impose high computational demands. To address this issue, we employed a single-target tracking strategy to ensure continuous tracking of pigs with ear tag loss. For potential multi-target requirements in production, multiple single-target trackers were executed in parallel to meet management needs.

**Figure 5:**
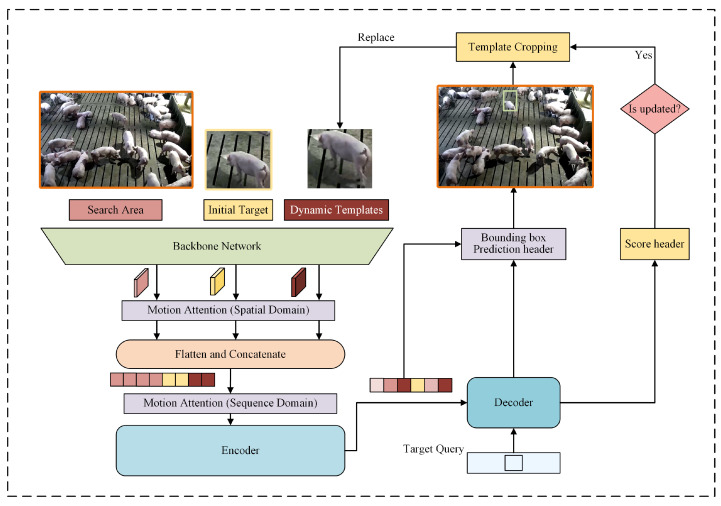
Architecture of the STARK-MOT tracker. The tracker consists of six components. The backbone network extracts features from the initial target, the search area, and dynamic templates. The feature pre-encoding module embeds Motion Attention to perform motion weighting in both spatial and temporal domains, producing motion-enhanced feature sequences. The encoder models relationships among features, while the decoder generates target queries. The prediction head performs bounding box localization and confidence scoring. Finally, the training and inference process updates dynamic templates to maintain robust tracking performance in production scenarios.

In actual production environments, the high visual similarity among breeding pigs complicates target differentiation during tracking. To address this challenge, we evaluated mainstream single-target tracking methods, such as SiamRPN++ [28] and MixFormer [29], and found that their robustness in complex scenarios—such as pose deformation and partial occlusion—remained insufficient. In contrast, STARK, based on the Transformer architecture [30], overcame these limitations by capturing global target features through the self-attention mechanism. Furthermore, to mitigate the effect of rapid pig movements on tracking accuracy in production, we proposed a Motion Attention-enhanced tracker, termed STARK-MOT, for pigs with ear tag loss, whose structure is shown in Figure 5. The model consists of six main modules: backbone network, feature pre-encoding, encoder, decoder, prediction head, and training and inference. By embedding Motion Attention to perform motion weighting in both spatial and temporal domains, the model’s ability to track fast-moving targets was significantly improved, leading to enhanced accuracy.

**Figure 6:**
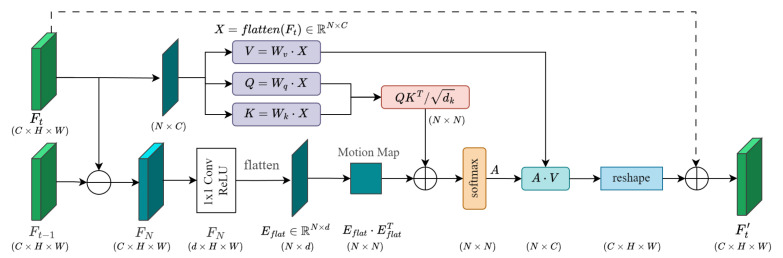
Architecture of the Motion Attention. The Motion Attention module computes the difference between the current and previous frame features to generate a motion map, which guides the attention mechanism, and applies motion weighting to the feature maps. Solid lines indicate the main data flow, and dashed lines represent residual connections.

(1) Backbone Network: STARK-MOT employed ResNet-50 [31] as its backbone. It took three inputs: (a) the initial target template of a calibrated breeding pig with ear tag loss obtained through the dual-view synergistic system, (b) the search region of the current frame representing the global visual image, and (c) a dynamically updated template sampled from intermediate frames. The backbone extracted hierarchical visual features from low to high levels, generating three sets of deep feature maps. The dynamic template was initialized with the calibrated pig with ear tag loss and was continuously refined during training and inference to improve tracking robustness.

(2) Feature Pre-encoding: The three sets of feature maps produced by the backbone were first compressed using a 1×1 convolution in the bottleneck layer. Subsequently, these feature maps are motion-weighted in the spatial domain through the Motion Attention module. The resulting feature maps were flattened and concatenated into three feature sequences along the spatial dimension. Then, the sequences underwent another round of motion weighting via the Motion Attention module in the sequence domain, enabling the encoder to focus more effectively on the target’s motion features. The structure of the Motion Attention module is illustrated in Figure 6.

To highlight significant motion regions within a frame, Motion Attention computed the element-wise residuals of the salient features from adjacent frames, as shown in Equation (5).

(5) $$D_{t}=\left|F_{t}-F_{t-1}\right|$$

In the equation, *t* represents the time step index; $F_{t}$ and $F_{t-1}$ are the feature maps output by the backbone in the current and previous frames, respectively.

Subsequently, channel compression and nonlinear transformation were applied to $D_{t}$ using a 1×1 convolution and ReLU, yielding $E_{t}\in R^{d\times H\times W}$. $E_{t}$ was then flattened into $E_{t}^{\mathrm{flat}}\in R^{N\times d}$. The spatial-domain motion guidance matrix was constructed through Equation (6).

(6) $$M_{s}=\mathrm{softmax}\left(E_{t}^{\mathrm{flat}}{\left(E_{t}^{\mathrm{flat}}\right)}^{⊤}\right)$$

where N=H·W, and $E_{t}^{\mathrm{flat}}{\left(E_{t}^{\mathrm{flat}}\right)}^{!⊤}$ denotes the pairwise position similarity matrix obtained through low-dimensional motion embedding.

In the attention computation, the spatial-domain motion guidance matrix was incorporated as a score bias term to assign higher weights to regions of significant motion, as shown in Equation (7).

(7) $$A_{s}=\mathrm{softmax}\left(\frac{QK^{⊤}}{\sqrt{d_{k}}}+\alpha M_{s}\right),\;Y_{s}=A_{s}V,\;F_{t}^{\prime }=\mathrm{reshape}\left(Y_{s}\right)$$

Here, $X=\mathrm{flatten}(F_{t})$, and *Q*, *K*, and *V* are the query, key, and value obtained by linear mapping of *X*. $d_{k}$ is the scale factor of the key and query; $M_{s}$ is the spatial-domain motion guidance matrix with weight coefficient α; $Y_{s}$ is the weighted sum of *V*; after normalization and reshaping, $F_{t}^{\prime }$ was obtained.

Finally, the features $F_{t}^{\prime }$ from the template and search branches were flattened and concatenated with positional encoding. Motion Attention was then applied again to complete motion weighting in the sequence domain.

(3) Encoder: The encoder was composed of multiple Transformer encoder layers. Each layer first captured dependencies among positions in the feature sequence using a multi-head self-attention module. The resulting features were then transformed through a feedforward network with nonlinear mapping. To stabilize gradient propagation and prevent degradation of feature representation, residual connections and layer normalization were applied between submodules.

(4) Decoder: The decoder integrated multiple target queries with the encoder output feature sequences as inputs. Through self-attention and cross-attention mechanisms, the query information was fused with global spatiotemporal features, producing representation vectors for target boxes.

(5) Prediction Head: Based on the representation vectors generated by the decoder, a fully convolutional network composed of Conv-BN-ReLU modules was employed to generate corner probability maps, predict bounding box coordinates, and output binary confidence scores. These results were further used to determine whether the dynamic template should be updated.

(6) Training and Inference: During inference, if the predicted confidence exceeded a preset threshold, the corresponding bounding box region was cropped to generate a new dynamic template, which replaced the previous one. This process enabled the tracker to capture appearance changes of the target over time.

When multiple breeding pigs with ear tag loss were detected in the same frame, a separate STARK-MOT tracking process was initiated for each pig. Each process was assigned to a dedicated GPU, allowing independent operation. This approach preserved the high accuracy and frame-rate advantages of single-target tracking while enabling simultaneous tracking of multiple pigs with ear tag loss.

#### 2.2.4. Experimental Settings

The experiments were conducted on a high-performance server running Ubuntu 20.04, equipped with two Intel(R) Xeon(R) Gold 6137 processors and six NVIDIA GeForce RTX 3090 GPUs. The software environment was based on a deep learning framework that included Miniconda3, Python 3.10.11, CUDA 11.7, PyTorch 2.0.0, and MMTracking 0.14. For the detector, a stochastic gradient descent (SGD) optimizer was used with a momentum factor of 0.9 and an initial learning rate of 0.02. The intersection over union (IoU) thresholds for the three stages of the cascaded RCNN were set sequentially to 0.5, 0.6, and 0.7. The batch size was 36, and the training lasted for 100 epochs. For the tracker, the AdamW optimizer was adopted, with the learning rate, learning rate multiplication factor, and maximum gradient clipping initialized at 0.0001, 0.1, and 0.1, respectively. A stepwise decay strategy was used for learning rate adjustment. The batch size was 114, and training was performed for 150 epochs.

#### 2.2.5. Evaluation Indicators

The Cascade-TLDP detector was evaluated using bounding box mean average precision (Bbox mAP), instance segmentation mean average precision (Mask mAP), number of parameters (Params), computational complexity (FLOPs), and detection speed.

The performance of the dual-view synergistic method was assessed using four indicators: target matching accuracy, coverage, dual-view mapping accuracy, and rejection rate. Specifically, target matching accuracy was defined as the proportion of correctly mapped samples among all pigs with ear tag loss that initiated dual-view mapping. Coverage referred to the proportion of samples in which the normalized distance between the predicted detection box centers from the two views did not exceed the preset threshold, relative to all pigs with ear tag loss requiring mapping. Dual-view mapping accuracy measured the proportion of correctly mapped samples among all pigs with ear tag loss intended for mapping. Rejection rate denoted the proportion of samples excluded from mapping results because the center-point distance exceeded the normalized threshold during candidate box selection under the nearest-neighbor principle.

The STARK-MOT tracker was evaluated using success rate (Success), normalized precision (Norm precision), precision, and model size. Success rate measured the proportion of frames where the intersection over union (IoU) between predicted tracking boxes and ground-truth annotations exceeded a predefined threshold, reflecting the overall tracking capability. Normalized precision was defined as the average Euclidean distance between the centers of predicted and ground-truth bounding boxes, normalized by target size, thereby evaluating spatial alignment under scale variation. Precision calculated the average pixel-level distance between the centers of predicted and ground-truth boxes, reflecting fine-grained localization accuracy. The computational formulas for these three metrics are provided in Equations (8)–(10).

(8) $$\mathrm{Success}\left(\theta \right)=\frac{1}{N}\sum_{i=1}^{N}I\left(\mathrm{IoU}\left(b_{i},g_{i}\right)\geq \theta \right)$$

In Equation (8), *N* denotes the total number of frames used for evaluation. $b_{i}$ represents the predicted bounding box in the *i*-th frame, and $g_{i}$ is the corresponding ground-truth bounding box. $\mathrm{IoU}(b_{i},g_{i})$ denotes the intersection over union between $b_{i}$ and $g_{i}$, while θ is the IoU threshold (set to 0.5 in this study). I(·) is an indicator function that returns 1 if the condition inside the parentheses is satisfied, and 0 otherwise.

(9) $$\mathrm{Norm}\_\mathrm{Precision}=\frac{1}{N}\sum_{i=1}^{N}\sqrt{\frac{{\left(x_{i}-x_{i}^{\prime }\right)}^{2}+{\left(y_{i}-y_{i}^{\prime }\right)}^{2}}{A}}$$

In Equation (9), $\left(x_{i},y_{i}\right)$ and $\left(x_{i}^{\prime },y_{i}^{\prime }\right)$ denote the center coordinates of the predicted and ground-truth bounding boxes in the *i*-th frame, respectively. *A* denotes the area of the image frame, serving as a normalization factor to ensure scale invariance.

(10) $$\mathrm{Precision}=\frac{1}{N}\sum_{i=1}^{N}\sqrt{{\left(x_{i}-x_{i}^{\prime }\right)}^{2}+{\left(y_{i}-y_{i}^{\prime }\right)}^{2}}$$

In Equation (10), the Euclidean distance between the predicted and ground-truth bounding box centers is computed for each frame, thereby quantifying the tracking error in pixel units.

## 3. Results

### 3.1. Pruning Performance Analysis of Cascade-TLDP Detector

We compared the impact of different sparsity rates on model performance under a fixed pruning rate of 0.5, with results summarized in Table 2. The model achieved the highest accuracy when the sparsity rate was set to 0.005.

**Figure 7:**
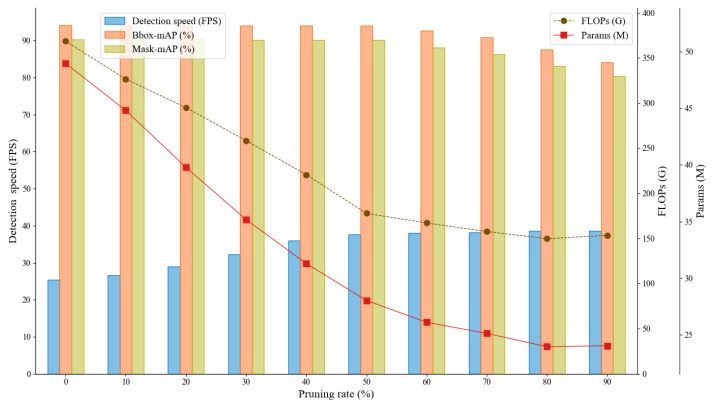
Performance analysis of the model under different pruning rates.

After fixing the sparsity rate at 0.005, we conducted lightweight experiments under different pruning rates. Figure 7 illustrates the trends of Bbox mAP, Mask mAP, parameter count (Params), FLOPs, and detection speed.

The results revealed that increasing the pruning rate gradually reduced both parameter count and computational complexity. Detection speed increased to 37.71 FPS when the pruning rate exceeded 0.5, although the growth rate slowed thereafter. A marked decline in accuracy was observed when the pruning rate exceeded 0.5. Considering all metrics, a pruning rate of 0.5 provided the best trade-off between model size and detection accuracy, and was therefore adopted in subsequent experiments.

### 3.2. Training Convergence Analysis of Cascade-TLDP and STARK-MOT

**Figure 8:**
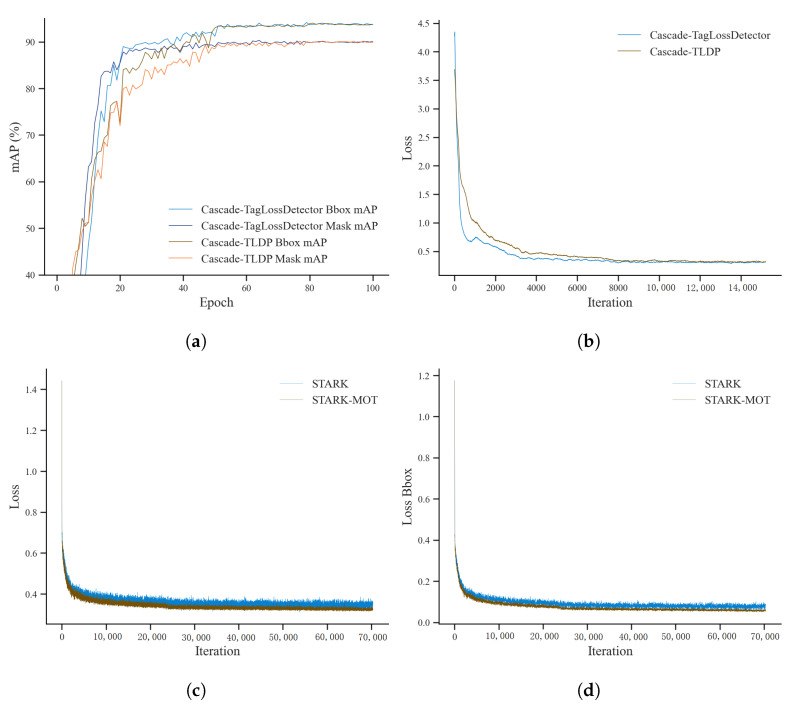
Training convergence curves of Cascade-TLDP and STARK-MOT. (**a**) Bbox mAP of Cascade-TLDP; (**b**) Mask mAP and Loss of Cascade-TLDP; (**c**) tracking loss of STARK and STARK-MOT; (**d**) bounding box loss of STARK and STARK-MOT.

Experiments were conducted on both the detection dataset and the tracking dataset, enabling the independent training of Cascade-TLDP and STARK-MOT. During training, the variations of Bbox mAP, Mask mAP, and overall loss for Cascade-TLDP, as well as Bbox loss and overall loss for STARK-MOT, were recorded to evaluate model performance, as illustrated in Figure 8.

From Figure 8a,b, it can be observed that the Bbox mAP, Mask mAP, and loss of Cascade-TLDP converge rapidly during the early training stages, showing trends highly consistent with those of Cascade-TagLossDetector. This indicates that the integration of channel pruning algorithms preserves the detection accuracy of the model. Specifically, Cascade-TLDP achieves convergence at approximately 94% for Bbox mAP and 90% for Mask mAP, in agreement with the detection results on the test set. This consistency confirms that the model does not suffer from overfitting or underfitting. As shown in Figure 8c,d, both STARK and STARK-MOT exhibit a rapid decline in bounding box loss and overall tracking loss during the initial training phases. With further iterations, the loss curves of STARK-MOT become smoother and stabilize at lower values compared with those of STARK, reflecting improved convergence. These results demonstrate that the introduction of the Motion Attention module effectively enhances the model’s temporal modeling capability, thereby improving its robustness and accuracy in tracking tasks.

### 3.3. Detection Results of Cascade-TLDP

To comprehensively evaluate the effectiveness of the proposed Cascade-TLDP in detecting breeding pigs with ear tag loss, we conducted a detailed comparison between Cascade-TLDP, Cascade-TagLossDetector and the baseline Cascade Mask R-CNN [32]. The evaluation is based on multiple performance indicators calculated on the test set, including bounding box mean average precision (Bbox mAP), instance segmentation mean average precision (Mask mAP), parameter count (Params), floating point operations (FLOPs), and detection speed. These metrics collectively reflect the models’ detection accuracy, computational complexity, and real-time performance, as summarized in Table 3.

Analyzing Table 3 showed that, compared with Cascade-TagLossDetector, the Bbox mAP and Mask mAP of Cascade-TLDP decreased by only 0.12 and 0.16 percentage points, respectively. However, the number of parameters and computational requirements were reduced by 42.74% and 41.93%, leading to a 48.87% improvement in detection speed. Compared with Cascade Mask R-CNN, Cascade-TLDP achieved gains of 2.89% in Bbox mAP, 2.53% in Mask mAP, and 16.07 fps in detection speed, while reducing parameters by 28.89 M and FLOPs by 183.28 G. Overall, Cascade-TLDP demonstrated clear advantages in accuracy, efficiency, and real-time performance. These improvements highlight the practical value of the proposed model for timely and accurate identification of breeding pigs with ear tag loss under real-world farming conditions, while ensuring feasibility for deployment on resource-constrained edge devices.

To further validate the detection performance of the proposed model, Cascade-TLDP was compared with several mainstream instance segmentation models. The results are summarized in Table 4.

As shown in Table 4, Cascade-TLDP achieved a favorable balance among accuracy, model size, and inference speed. It attained a Bbox mAP of 94.03% and a Mask mAP of 90.16%, outperforming YOLO11x-seg, Mask2Former, and OneFormer, which highlights its superior capability in pig body detection and mask segmentation. Moreover, Cascade-TLDP contained only 28.04 M parameters with a computational load of 177.81 G, while delivering an inference speed of 37.71 fps. This speed substantially exceeded that of the other methods, underscoring its advantage in real-time monitoring scenarios. Although Mask DINO achieved a slightly higher Mask mAP than Cascade-TLDP, its high computational cost and slow inference severely limited its suitability for real-time applications. In contrast, Cascade-TLDP simultaneously reduced computational complexity, improved inference speed, and maintained high detection accuracy, making it particularly suitable for deployment in large-scale farming environments. These findings demonstrate its potential as an efficient and reliable technical solution for monitoring breeding pigs with ear tag loss.

### 3.4. Effect Verification of the Dual-View Synergistic Method

**Figure 9:**
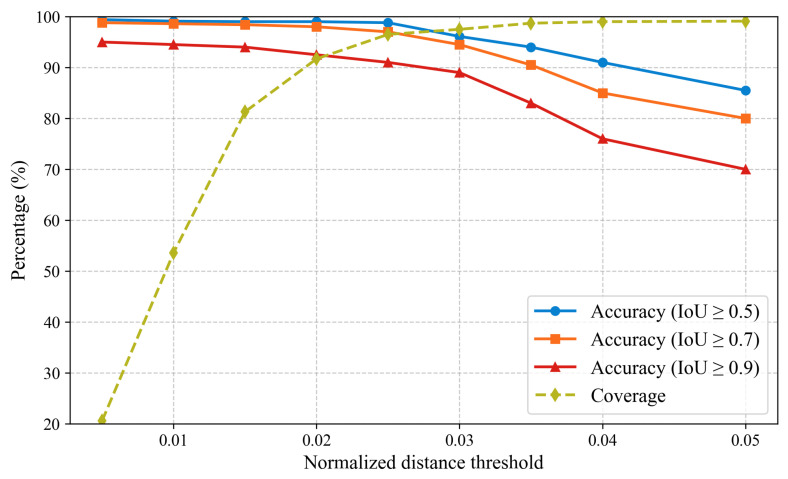
Impact of normalized distance threshold on the performance of dual-view position mapping.

To evaluate the effectiveness of the proposed dual-view synergistic method in mapping the locations of breeding pigs with ear tag loss, coverage and target matching accuracy were analyzed under different normalized distance thresholds. As shown in Figure 9, coverage increased monotonically with the rise of the normalized distance threshold. When the threshold exceeded 0.03, coverage stabilized at approximately 98%, indicating that most ear tag loss pig samples could be successfully mapped and matched. At the same time, target matching accuracy under a fixed intersection over union (IoU) standard gradually declined as the distance threshold increased. This suggests that while a larger threshold improves the overall identification rate, it also raises the risk of mismatches. Furthermore, under the same distance threshold, higher IoU requirements corresponded to lower accuracy, as stricter overlap constraints increased the difficulty of matching. Overall, when the normalized distance threshold was set to 0.025 and IoU to 0.5, the method achieved the best trade-off, with an accuracy of 95.3%.

Comparative experiments were further conducted under the optimal normalized distance threshold and IoU settings across four scenarios: daytime stationary, daytime moving, nighttime stationary, and nighttime moving. The results are presented in Table 5. In daytime stationary scenes, dual-view mapping achieved the highest accuracy (95.9%) with the lowest normalized center error, confirming the robustness of the method under favorable conditions. In contrast, nighttime moving scenes, affected by motion blur and low illumination, exhibited slightly lower accuracy and positioning precision; nevertheless, performance remained high overall. The rejection rate across all scenarios was maintained between 3.0% and 4.7%, demonstrating that the system effectively avoids mismatches while ensuring stable mapping performance.

### 3.5. Performance of Target Tracking

**Figure 10:**
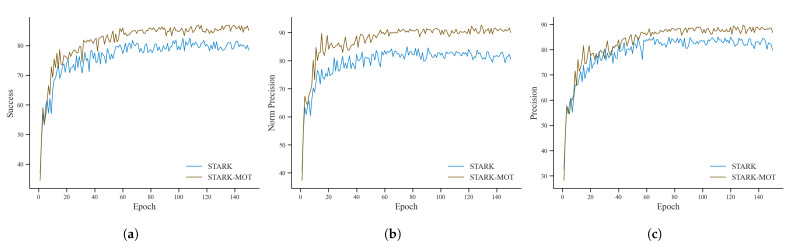
Comparison of STARK and STARK-MOT tracking performance during training. (**a**) Success rate; (**b**) normalized precision; (**c**) precision.

Figure 10 compares the success rate, normalized precision, and precision of STARK and STARK-MOT in the pig tracking task on the training set.

The performance metrics exhibited a consistent upward trend throughout training. After approximately 60 epochs, the success rate, normalized precision, and precision stabilized and eventually converged at 87%, 92%, and 89%, respectively. These convergence values were highly consistent with the results obtained on the test set, thereby validating the training procedure and confirming the generalization capability of the model. Moreover, STARK-MOT consistently outperformed the original STARK model across all three evaluation metrics, indicating that the integration of the Motion Attention module substantially improved tracking continuity, spatial localization accuracy, and overall robustness under complex and dynamic conditions.

The comparative performance of STARK-MOT and the baseline STARK model on the test set is summarized in Table 6. Specifically, STARK-MOT achieved a success rate of 86.91%, a normalized precision of 92.68%, and a precision of 89.74%, representing improvements of 4.39, 3.22, and 4.77 percentage points, respectively, over the original STARK model. These results clearly demonstrate that embedding the Motion Attention mechanism into the STARK framework significantly enhanced tracking performance in terms of both accuracy and robustness.

Moreover, the introduction of Motion Attention adds only marginal computational overhead. The model size increases by just 2.84 million parameters, without introducing additional structural complexity or inference delays. This lightweight design ensures that STARK-MOT remains suitable for end-to-end training and real-time deployment. The seamless integration of Motion Attention into the existing framework further demonstrates its practicality and scalability. Overall, STARK-MOT not only achieves superior tracking accuracy but also maintains high computational efficiency, underscoring its strong potential for deployment in resource-constrained or edge-computing environments typical of intelligent livestock farming. In addition, unlike multi-target tracking models, single-target tracking inherently reduces the risk of mismatches during the correlation phase. This is particularly advantageous in scenarios where individuals are monitored separately, as it effectively suppresses identity switching caused by occlusion or group interactions.

### 3.6. Potential Application of STARK-MOT

**Figure 11:**
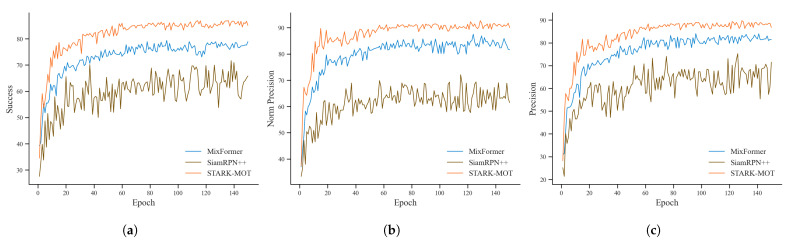
Comparison of tracking performance during training between STARK-MOT, SiamRPN++, and MixFormer. (**a**) Success rate; (**b**) normalized precision; (**c**) precision.

To further evaluate the practical potential of the proposed model in real-world production environments, comparative experiments were conducted against SiamRPN++ and MixFormer on the training set. As illustrated in Figure 11, STARK-MOT converged faster and exhibited smoother and more stable growth in success rate, normalized precision, and precision. All three metrics ultimately reached the highest levels among the compared methods, demonstrating the superior tracking performance of STARK-MOT.

Table 7 presents a comparative analysis of tracking performance under different lighting conditions. On the daytime dataset, STARK-MOT achieved a success rate of 87.36%, a normalized precision of 93.24%, and a precision of 90.09%, outperforming both MixFormer and SiamRPN++ by a clear margin. These results highlight the strong tracking capability of STARK-MOT under favorable lighting conditions.

In the nighttime dataset, all three models showed performance degradation due to reduced visibility and weaker appearance features. However, STARK-MOT experienced the smallest decline, with decreases of only 1.44% in success rate, 1.37% in normalized precision, and 0.78% in precision. This moderate reduction demonstrates the model’s strong generalization capability under low-light conditions. The stability of STARK-MOT can be attributed to the Motion Attention mechanism, which leverages temporal continuity and inter-frame motion cues to compensate for diminished visual information. These findings underscore the practical applicability of STARK-MOT for robust tracking of breeding pigs with ear tag loss in production environments.

## 4. Discussion

**Figure 12:**
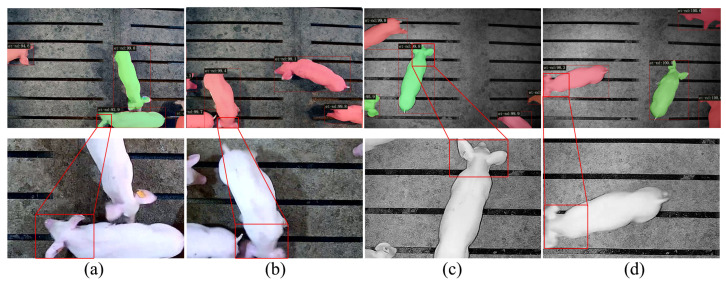
Detection results of Cascade-TLDP across different production scenarios.

This paper introduces channel pruning into the Cascade-TLDP detector to selectively eliminate redundant channels within the backbone network. With a pruning rate of 0.5 and a sparsity rate of 0.005, the model achieves a reduction of 20.93 M parameters, a decrease of 128.4 G in computational complexity, and an approximate 49% improvement in detection speed. In terms of detection accuracy, Cascade-TLDP successfully identified pigs across diverse scenarios. On the test set, which contained 517 labeled breeding pigs, the model accurately detected 479, yielding a detection rate of 92.69%. The model’s performance in complex scenes is illustrated in Figure 12, where pink denotes breeding pigs with invisible ear tags and green indicates those with visible ear tags. Cascade-TLDP effectively distinguishes between the two categories. In Figure 12a,b representing typical daytime scenarios, the model accurately identifies and segments targets despite ear tag occlusion and motion blur. In more challenging environments, such as low-light conditions (Figure 12c) and nighttime motion blur (Figure 12d), the model still reliably detects and classifies individual pigs.

The dual-view synergistic method proposed in this study addresses the limitations of single-view detection by enabling positional mapping of breeding pigs with ear tag loss from multiple perspectives in real farm environments. Experimental results show that this method substantially improves spatial coverage, achieving a localization accuracy of 95.3%. This improvement is largely attributed to the introduction of a normalized distance threshold and a nearest-neighbor priority control strategy. During target matching, the system selects the most probable individual based on the nearest-neighbor principle, while filtering out low-confidence candidates with significant positional deviations through the distance threshold. This approach effectively reduces mismatching and strikes a balance between coverage and accuracy in complex scenarios such as pig clustering, occlusion, and rapid movement. The findings are consistent with the multi-view 3D coordinate and feature fusion scheme proposed by Huo et al. (2022) [33], both demonstrating that cross-view feature matching and spatial mapping can enhance localization accuracy under challenging conditions.

**Figure 13:**
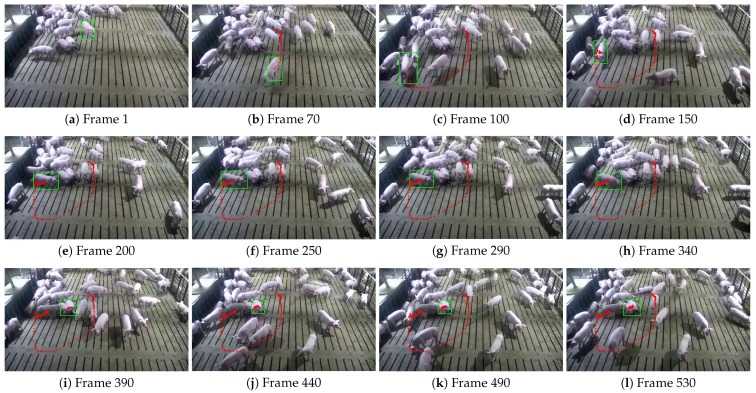
Tracking performance of the STARK model on daytime datasets. The green box indicates the detected position of the pig, while the red dots represent its walking trajectory.

**Figure 14:**
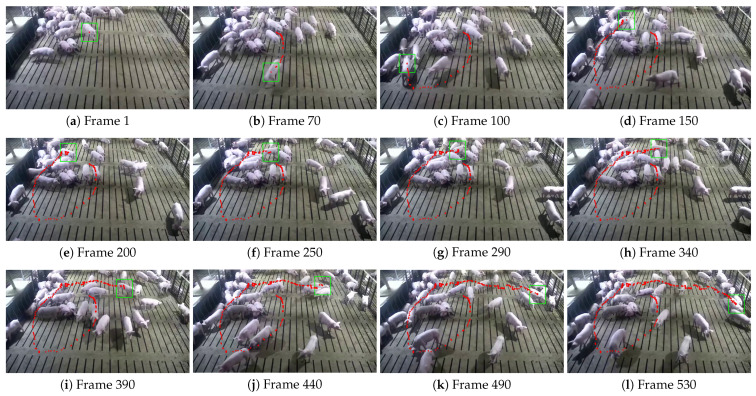
Tracking performance of the STARK-MOT model on daytime datasets. The green box indicates the detected position of the pig, while the red dots represent its walking trajectory.

This study further visualized the trajectories of pigs with ear tag loss using the central coordinates of their bounding boxes, comparing the tracking results of the STARK model and the proposed STARK-MOT model (Figure 13 and Figure 14). In the visualization, green rectangular boxes represent detected breeding pigs, while red dots indicate their movement trajectories.

Figure 13 and Figure 14 present the tracking visualization results of the baseline STARK model and the proposed STARK-MOT model, respectively, on the daytime dataset for breeding pigs with ear tag loss using interval-sampled frames. As shown in Figure 13, STARK exhibits noticeable tracking drift in multiple frames. In particular, position deviations are observed in frames (i)–(l), while frame (d) demonstrates a complete target loss, resulting in an interrupted trajectory. These failures are mainly caused by rapid movement and partial occlusion during group interactions, which challenge the global self-attention-based feature matching strategy of STARK and hinder its ability to maintain stable tracking. In practical applications, such instability could result in the omission or misclassification of key behaviors, thereby reducing the reliability and effectiveness of automated monitoring systems. In contrast, as shown in Figure 14, the proposed STARK-MOT exhibits markedly improved temporal consistency and tracking robustness under the same conditions. Even in the presence of occlusion, abrupt motion, and pose variation, STARK-MOT consistently maintains accurate target localization and generates continuous, coherent trajectories without evident drift.

**Figure 15:**
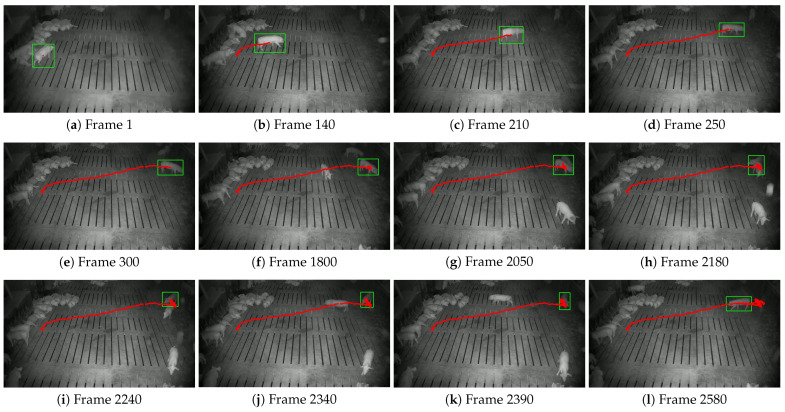
Tracking performance of the STARK model on nighttime datasets. The green box indicates the detected position of the pig, while the red dots represent its walking trajectory.

**Figure 16:**
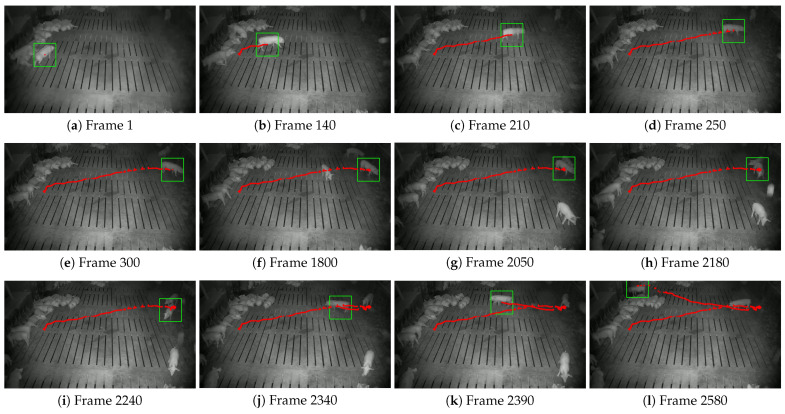
Tracking performance of the STARK-MOT model on nighttime datasets. The green box indicates the detected position of the pig, while the red dots represent its walking trajectory.

As illustrated in Figure 15, the tracking stability of STARK deteriorates significantly under low-illumination conditions due to reduced image contrast and weakened appearance features at night. The model exhibits evident drift across several key frames, particularly in frames (h)–(j), where the predicted bounding boxes deviate from the actual targets, and the generated trajectories show jittering before ultimately breaking off. These issues indicate that STARK struggles to capture long-term temporal dependencies in complex, low-light scenarios. In contrast, Figure 16 demonstrates that STARK-MOT maintains superior robustness and temporal continuity under the same nighttime conditions. Despite challenges such as blurred appearance, insufficient lighting, and partial occlusion, STARK-MOT consistently outputs accurate target positions and maintains smooth, uninterrupted trajectories. Throughout the entire sequence, no significant drift is observed.

The results demonstrate that the introduced Motion Attention module plays a critical role in temporal sequence modeling. By effectively capturing both motion cues and positional changes between frames, the module enables STARK to prioritize motion features during multi-frame feature encoding. This substantially enhances the model’s capacity to recover targets and improves discrimination stability in complex dynamic scenes, thereby allowing for more accurate reconstruction of the motion trajectories of breeding pigs with ear tag loss.

At present, the detection and localization of ear tag loss breeding pigs in commercial farms primarily depend on manual observation, which suffers from limited timeliness, high labor demands, and increased animal stress. Although some farms employ RFID ear tags and passage-based devices, these approaches require animals to move through designated checkpoints, which hinders real-time monitoring across the entire facility. In contrast, the dual-view synergistic method proposed in this study achieves real-time detection, precise localization, and continuous tracking of breeding pigs with ear tag loss by deploying two cameras for 24-h video acquisition, combined with lightweight detectors and trackers. This approach markedly improves automation and timeliness, reduces the risk of individual identity confusion caused by ear tag loss, enhances management efficiency, promotes animal welfare, and has the potential to mitigate economic losses.

Despite these promising results, certain limitations remain. First, the generalization ability of the model requires further validation, as all experimental data were collected from a single farm. Differences in barn layouts, lighting conditions, and camera installation practices across farms may affect stability and accuracy. Second, the robustness of the system under extreme conditions—such as lens contamination or frame loss during video transmission—has not been systematically assessed. To address these challenges, future research will focus on collecting data from diverse scenarios, growth stages, and production models to construct a more comprehensive dataset. Data augmentation and model optimization strategies will also be adopted to further improve generalization performance. In addition, integrating multi-modal information, including infrared thermal imaging and depth data, will be explored to enhance robustness under complex interference conditions, thereby supporting stable deployment and long-term operation in large-scale, real-world farming environments.

## 5. Conclusions

This study addresses the challenge of individual identity loss or confusion caused by ear tag loss in breeding pigs. A dual-view synergistic detection and tracking method is proposed to support production managers in promptly identifying ear tag loss events, accurately locating affected pigs, and reapplying replacement tags in a timely manner. Specifically, two cameras—providing localized top-down and panoramic oblique views—are deployed to collect continuous video data. The lightweight Cascade-TLDP model detects ear tag loss pigs from the localized top-down view, after which a dual-view synergistic mapping method determines their positions in the panoramic view. Finally, the STARK-MOT model enables continuous and robust tracking. Experimental results demonstrate that the proposed non-invasive approach achieves high-precision detection and stable tracking of ear tag loss pigs under real production conditions, thereby offering effective technical support for automated monitoring and identity management in precision livestock farming. Furthermore, the method exhibits strong scalability and can be extended to cattle, sheep, and other livestock production systems, contributing to improved efficiency in precise animal husbandry management.

## Figures and Tables

**Table 1:** Distribution of datasets for detecting and tracking pigs with ear tag loss.

Conditions	Movement State	Ear Tag Loss Detection Dataset			Individual Tracking Dataset				
		**Training Set**	**Test Set**	**Images**	**Training Video**	**Training Trajectories**	**Test Video**	**Test Trajectories**	**Trajectories**
Daytime	Active state	1123	280	1403	368	1396	92	348	1744
	Mixed state	1690	422	2112					
	Stationary state	1515	378	1893					
Nighttime	Active state	251	62	313	258	980	64	244	1224
	Mixed state	438	109	547					
	Stationary state	387	97	484					
Overall	Active state	1374	342	1716	626	2376	156	592	2968
	Mixed state	2128	531	2659					
	Stationary state	1902	475	2377					

**Table 2:** Model performance under different sparsity rates at a pruning rate of 0.5.

Pruning Rate	Sparsity Rate	Bbox mAP (%)	Mask mAP (%)	Recall (%)
0.5	0.0001	90.64	85.39	89.24
0.5	0.0025	93.47	88.26	91.89
0.5	0.005	94.03	90.16	92.71
0.5	0.01	93.62	89.09	91.08

**Table 3:** Comparison of the detection performance of Cascade-TLDP, Cascade Mask R-CNN, and Cascade-TagLossDetector.

Method	Bbox mAP (%)	Mask mAP (%)	Params (M)	FLOPs (G)	Detection Speed (fps)
Cascade Mask R-CNN	91.14	87.63	56.93	361.09	21.64
Cascade-TagLossDetector	94.15	90.32	48.97	306.21	25.33
Cascade-TLDP	94.03	90.16	28.04	177.81	37.71

**Table 4:** Comparison of detection performance with mainstream instance segmentation methods.

Method	Bbox mAP (%)	Mask mAP (%)	Params (M)	FLOPs (G)	Detection Speed (fps)
Cascade-TLDP	94.03	90.16	28.04	177.81	37.71
YOLO11x-seg	92.16	89.27	62.10	319.21	27.28
Mask DINO	92.68	90.71	115.23	351.97	12.30
Mask2Former	91.32	88.59	212.33	375.16	8.40
OneFormer	91.10	88.22	219.75	436.84	6.80

**Table 5:** Performance analysis of dual-view position mapping in different scenarios.

Scenario	Accuracy (%)	Coverage (%)	Dual-View Mapping Accuracy (%)	Rejection Rate (%)
Daytime, Stationary	98.9	97.0	95.9	3.0
Daytime, Active	98.5	96.2	94.8	3.8
Nighttime, Stationary	98.2	96.0	94.3	4.0
Nighttime, Active	97.8	95.3	93.2	4.7
Overall	98.8	96.5	95.3	3.5

**Table 6:** Performance comparison of STARK and STARK-MOT on the test set.

Method	Success (%)	Norm Precision (%)	Precision (%)	Detection Speed (fps)	Model Size (M)
STARK	82.52	89.46	84.97	27.88	107.23
STARK-MOT	86.91	92.68	89.74	26.02	110.07

**Table 7:** Tracking performance of STARK-MOT, SiamRPN++, and MixFormer under different lighting conditions.

Conditions	Model	Success (%)	Norm Precision (%)	Precision (%)
Daytime	MixFormer	81.01	88.72	85.32
	SiamRPN++	71.90	72.26	75.95
	STARK-MOT	87.36	93.24	90.09
Nighttime	MixFormer	75.29	86.05	81.66
	SiamRPN++	70.97	71.84	74.53
	STARK-MOT	85.92	91.87	89.31
Overall	MixFormer	79.40	87.51	84.02
	SiamRPN++	71.67	72.08	75.26
	STARK-MOT	86.91	92.68	89.74

## Data Availability

The datasets presented in this article are not readily available because the data are part of an ongoing study. Requests to access the datasets should be directed to the corresponding author.
